# Comments on "Radiological perspective on earthquake trauma: differences in children and adults"

**DOI:** 10.1590/1806-9282.20241899

**Published:** 2025-06-02

**Authors:** Gokhan Tonkaz, Tumay Bekci

**Affiliations:** 1 Giresun University, Faculty of Medicine, Department of Radiology – Giresun, Turkey.

Dear Editor,

We read with a great deal of interest the article titled "Radiological perspective on earthquake trauma: differences in children and adults"^1^. This study provides valuable insights into earthquake-related injuries in both pediatric and adult populations. However, we believe that several aspects of the study require further discussion and clarification.

First, the authors categorized earthquake-related injuries into seven groups (cerebral, spinal, maxillofacial, thoracic, abdominal, pelvic, and extremity). However, they did not account for sub-age groups and imaging intervals. In our research, head injuries were more common in children aged 0–4 years, while rib fractures and spinal and pleural injuries were more prevalent in the 15–18 years age group^2^. Among adults, spinal injuries were slightly more frequent in individuals over 65 years of age, likely due to decreased bone density in this population^3^. It is evident that, beyond differences between children and adults, there are significant variations in injury types across sub-age groups in both populations due to differing injury mechanisms. A more detailed analysis of the relationship between sub-age groups and injury types would strengthen the findings of the study.

Second, the authors indicate that "due to the history of widespread crush injuries," the use of contrast agents was minimized. However, as this was a retrospective study, they did not provide details on how or to what extent this was implemented, nor on the specific considerations taken into account. It is unclear how the conclusion was reached that the use of contrast agents was minimized in patients transferred from the chaotic environment caused by the earthquake, especially when it is uncertain whether patient records were adequately maintained.

Third, in the study, patients who underwent fasciotomy surgery based on clinical history and physical examination findings indicative of compartment syndrome were classified as having compartment syndrome. In a retrospective study where patients’ clinical conditions were noted as unknown and magnetic resonance imaging (MRI) was not utilized, relying solely on fasciotomy as the criterion for diagnosing compartment syndrome could lead to underdiagnosis. Patients who died without undergoing surgery may have been overlooked. In a radiology-based study, including a diagnosis like compartment syndrome, which relies heavily on clinical findings, may not provide significant added value.

Fourth, we appreciate the authors’ acknowledgment of the study's limitations that the absence of MRI in the diagnostic process is a disadvantage. In a study based on computed tomography and plain radiographs, the lack of MRI utilization in earthquake-related injuries may have resulted in missed diagnoses of conditions, such as diffuse axonal injury. Undoubtedly, the use of MRI in selected patients would provide significant benefits for patient management. This represents a fundamental limitation of the study.

Finally, we hope that the findings of this study will contribute to the development of guidelines and disaster preparedness for future major earthquakes^3,4^. We believe that this topic warrants further discussion and investigation.
